# Probe-Based Confocal Laser Endomicroscopy Evaluation of Colon Preneoplastic Lesions, with Particular Attention to the Aberrant Crypt Foci, and Comparative Assessment with Histological Features Obtained by Conventional Endoscopy

**DOI:** 10.1155/2012/645173

**Published:** 2012-04-08

**Authors:** Massimo Mascolo, Stefania Staibano, Gennaro Ilardi, Maria Siano, Maria Luisa Vecchione, Dario Esposito, Gaetano De Rosa, Giovanni Domenico De Palma

**Affiliations:** ^1^Department of Biomorphological and Functional Sciences, Pathology Section, University of Naples “Federico II”, 80131 Naples, Italy; ^2^Center of Excellence for Technical Innovation in Surgery (ITC), Department of General Surgery, Geriatrics, Oncology, and Advanced Technology, University of Naples “Federico II”, 80131 Naples, Italy; ^3^Oncology Research Center of Basilicata, CROB, 85028 Rionero in Vulture, Italy

## Abstract

The colorectal carcinoma represents one of the most common and aggressive malignancies, still characterized by an unacceptable mortality rate, mainly due to the high metastatic potential and to a late diagnosis. In the last years, the research community focused on the chance of improving the endoscopic screening to detect neoplastic lesions in a very early stage. Several studies proposed aberrant colonic crypt foci as the earliest recognizable step of transformation in colonic multiphase carcinogenesis. We previously demonstrated the clinical applicability and predictive power of probe-based confocal laser endoscopy (pCLE) in superficial colorectal neoplastic lesions and also characterized *in vivo* a case of dysplasia-associated lesional mass (DALM) in ulcerative colitis. Now, we aim to evaluate the accuracy of pCLE in the detection of ACF comparing in double-blind manner the microendoscopic and histopathological features resulting from colonic biopsy. By pCLE, we identified specific crypt architecture modifications associated with changes in cellular infiltration and vessels architecture, highlighting a good correspondence between pCLE features and histology.

## 1. Introduction

Colorectal cancer represents the third most common human malignancy after prostate and lung cancer in males and the second one after breast carcinoma in females, with more than 1.200.000 new cases [1, 2], constituting a major cause of cancer death worldwide, particularly in Europe where it is responsible for more than 200.000 deaths per year [3]. Although this unacceptable mortality rate is closely associated with high metastatic ability of colorectal cancer, many of the deaths are caused by a late diagnosis. In fact, the prognosis of each malignancy strongly depends on stage at diagnosis and most cancers can be successfully treated if diagnosed at an early stage. For this reason, the research community focused its efforts not only in attempt to better understand the molecular mechanisms underlying the colon carcinogenesis but also on the possibility to improve the endoscopic screening of the colorectal lesions in the very early stage. To date, colon endoscopy remains the best way to make cancer prevention of this district possible. Given the finding that conventional colonoscopy sometimes is not able to differentiate between neoplastic and nonneoplastic lesions, several studies evaluated the role of advanced new endoscopic imaging techniques, such as chromoendoscopy and confocal laser endomicroscopy (CLE), in the detection of colorectal lesions [4–6]. CLE enables to obtain *in vivo* microscopic images during endoscopy, allowing to make real-time adequate diagnosis and to perform target biopsies improving the diagnostic accuracy. Presently, there are two devices to perform CLE: the endoscope-based confocal laser endomicroscopy (eCLE; Pentax, Tokio, Japan), in which a confocal probe is incorporated in the tip of a routinary endoscope, and the probe-based confocal laser endomicroscopy (pCLE), in which the stand-alone probe can be passed through the biopsy channel of traditional endoscope (Cellvizio, Mauna Kea Technologies, Paris, France) [4, 5, 7]. To date there are not still adequate data to consider an endoscopic technique better than the other one.

It is known that colorectal carcinogenesis is a multistep process progressing through several morphological stages [8]. The earliest phase may be the formation of aberrant crypt foci (ACF). In fact, the ACF prevalence and density are greater in patients with colorectal carcinoma and adenoma, compared to normal controls, and, therefore, these lesions could be used as biomarker of colorectal cancer [9, 10]. Moreover, considering that ACF are preventable preneoplastic lesions and that their growth is modified by specific modulators, their early detection is very important [11]. However, there is still a wide variation of endoscopic criteria useful to identify and define ACF. The main considered feature is the mucosa color [12–15], usually darker compared to the adjacent normal colonic mucosa, but also the crypt architecture, the crypt lumen size [13, 14, 16], the raised appearance [13, 15, 16], and the thickness of epithelial lining [14] are considered. A height of less than 2 mm has been proposed to differentiate them from colonic polyps in some recent studies [17].

In a previous study, conducted as conclusion of MIUR/PRIN project (2007) on this specific topic, we demonstrated that pCLE constitutes a reliable tool for the identification of colorectal superficial carcinoma [4]. In addition, we first discussed the pCLE findings regarding a case of dysplasia-associated lesional mass (DALM) in chronic ulcerative colitis (CUC) [5]. On the basis of these previous reports, we correlate for the first time endoscopic and histological features of ACF in the attempt to validate the promising role of pCLE as useful and predictive tool of evaluation of colorectal preneoplastic lesions.

## 2. Materials and Methods

### 2.1. Patients

A small group constituted of 9 patients with evidence of ACF at routine colonoscopy were enrolled for this study. Endoscopic features considered for the identification of ACF, according to the literature data, were darker colonic mucosa after dying, the two-threefold crypt lumen size, raised appearance, and/or thickened epithelium [12–14, 16, 18]. Exclusion criteria were patients aged less than 18 years, known allergic diseases, and impaired renal function. In addition, 5 patients with colonic adenomas and 5 patients with adenocarcinoma at endoscopy were selected. Three hyperplastic polyps were added as control.

### 2.2. Equipment

Lesions were identified using white-light endoscopy and NBI followed by pCLE imaging recorded by a Coloflex UHD-type probe, using the Cellvizio Endomicroscopy System (Mauna Kea Technologies, Paris, France) [5]. Coloflex UHD-type probe is a 2.5 mm catheter probe inserted through the endoscope-working channel to obtain dynamic images of the mucosa. pCLE imaging data were registered at a scan rate of 12 frames per second with a scanning field of 30,000 pixels. The field of view is of 240 × 200 *μ*m, with a lateral resolution of 1 *μ*m. From single video frames is reconstructed 1 larger static image (4 × 2 mm) by a special computer software (mosaicing), which uses a hierarchical framework algorithm able to recover a globally consistent alignment of the input frames, to compensate for motion-induced distortions and to capture nonrigid deformations. The resulting image combines all moving images, cancels motion artifacts, and reconstitutes panoramas of the tissues.

### 2.3. Procedure

All examinations were performed by a single experienced endoscopist (GDDP). During twenty-four hours before the procedure, 4 L of isotonic polyethylene glycol solution was administered as a bowel cleansing. A conscious sedation with midazolam (5–10 mg i.v.) was administered when requested by the patient. After the identification of each lesion on white-light endoscopy or NBI, a 10–20 mg intravenous bolus of Buscopan (hyoscine-N-butyl-bromide) was given to limit peristaltic artifacts, followed by the administration of 10 mL of 10% sodium fluorescein for CLE image acquisition. Confocal images of circumscribed lesions and four segmental “normal” colorectal quadrants were acquired, the latter used to define normality. Specimens obtained (resected lesions and/or target biopsy) were formalin fixed and paraffin embedded and, then, stained with hematoxylin-eosin. The histologic evaluation was performed by two experienced pathologists (MM and SS) in a blinded fashion and graded in accordance with the Vienna modified classification of gastrointestinal epithelial neoplasia [19]. Histologically, ACF were defined as enlarged crypts (at least 1.5 times larger than normal), covered by thickened epithelium with lack of stratification, but characterized by regular nuclei with only mild or focal crowding, often elevated from adjacent normal mucosa, according to the proposed criteria [20–22].

### 2.4. Main Outcome Measurements

According to the Paris Workshop guidelines [23], all identified lesions were classified as follows: protruding lesions (Ip: pedunculated polyp; Ips: subpedunculated polyp; Is: sessile polyp); flat elevated lesions (0-IIa: flat elevation of mucosa; 0-IIa/c: flat elevation with central depression); flat lesions (0-IIb: flat mucosal change; 0-IIc: mucosal depression; 0-IIc/IIa: mucosal depression with raised edge). The diagnostic endoscopic criteria used for diagnosing ACF were crypts larger in diameter than the surrounding normal crypts, from which they are distinguished by deeper color when stained with methylene blue, thicker epithelium and raised appearance [9, 12–14, 16, 24–27]. The endoscopy operator (GDDP) made a preliminary diagnosis based upon the *in vivo* images (video sequences) and the mosaic images, according to the Miami confocal endomicroscopy criteria for the prediction of intraepithelial colorectal neoplasia [28]. pCLE diagnosis was then compared with the histopathological diagnosis. Every image was judged as good, average, or poor by the principal investigator, basing on presence/absence of moving artifacts and on a well/poor recognizable crypt and vascular architecture. To assess interobserver agreement, 50 confocal video images and mosaicing images of good or average quality (25 images of neoplastic lesions and 25 images of nonneoplastic lesions or normal colorectal epithelium) were randomly selected and evaluated in a blinded fashion by one endoscopist (DE) with minimal experience with pCLE. Their prediction of malignant or benign features on pCLE was compared with the histopathologic diagnosis.

## 3. Results

For this study, 9 patients (4 males, mean age 65 years, range 56–83) with endoscopic evidence of ACF, 5 patients with colonic adenoma (5 males, mean age 60,6, range 49–72), 5 patients with colonic carcinoma (3 males, mean age 63,2, range 56–73) and 3 patients with hyperplastic polyps (2 males, mean age 57,7, range 41–76) were considered. A total of 30 lesions were identified. A single lesion was found in 14 (63,6%) cases, and 8 (36,4%) patients had two lesions. The lesions were located in the rectum in 5 cases, in the sigmoid colon in 4 cases, in the descending colon in 5 cases, and in the right colon in 8 cases.

### 3.1. Correlation of Histopathology and pCLE Images

On pCLE examination, normal mucosa was defined by a hexagonal, honeycomb appearance with a round crypt structure, surrounded by regular vessels, covered by a homogeneous epithelium with “black-hole” goblet cells in the subcellular matrix; hyperplastic mucosa was characterized by crypts with slit or stellate openings covered by uniform epithelium, with a regular vessel architecture, with some increase in pericryptic capillary density; neoplastic tissue was represented by “dark” cells, with mucin and goblet cell/crypt density depletion; the architectural pattern was irregular, as well as the epithelial thickness, with villiform structures or crypt fusion and distortion, and “dark” epithelial border. The blood vessels were dilated and irregularly branching.

A suspected ACF, identified with traditional endoscopy, can show characteristics of dysplastic adenoma or hyperplastic polyp on pCLE, as previously described.

**Figure 1:**
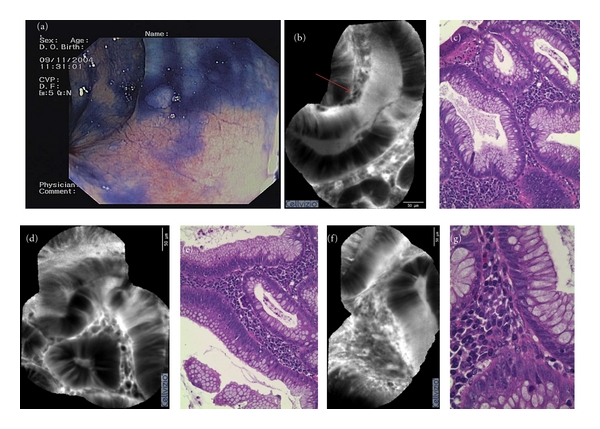
(a) Conventional “white-light” endoscopy of an ACF; (b), (d), and (f) pCLE of the lesion showing an enlarged crypt (red arrow, (b)) and normal globet cell density; (c), (e), and (g) hystologic features of the lesion, showing some enlarged crypts, with thickened epithelium with partial lack of stratification, in the presence of mild dysplasia.

All pCLE images diagnosed as “normal” mucosa showed normal architecture at histopathologic evaluation. Among cases recorded as ACF at pCLE, histologic evaluation confirmed the presence of aberrant crypts in 7 biopsy specimens and in two of these cases a diagnosis of microadenoma with low-grade displasia was made (Figure 1). Four out of 5 lesions diagnosed as adenoma and 5/5 diagnosed as adenocarcinoma at pCLE showed correspondence at histology. One lesion diagnosed as adenoma on CLE at histologic evaluation was diagnosed as hyperplastic polyp. Hyperplastic polyps used as control and so selected on confocal imaging were confirmed as benign on histology, but in 2 of these features of hyperplasia were showed. No patients showed endoscopic complications or adverse reactions to sodium fluorescein; only a slight yellowish discoloration of the skin was recorded, which usually disappeared within 30–60 min.

## 4. Discussion

Although to date there is not still a close correspondence between the conventional endoscopic images and histological assessment, this association of tools remains the best way to diagnose accurately and then treat as early as possible many diseases of different district, especially of the colorectal tract, including chronic inflammatory, preneoplastic, and neoplastic diseases. Basing upon the finding that conventional colonoscopy is not always able to differentiate between neoplastic and nonneoplastic lesions, in recent years, several studies highlighted the potential use of confocal laser endomicroscopy (CLE), a new emerging technique, in the screening patients for early colorectal cancer detection and prevention [4–6, 29, 30]. This technique allows to obtain *in vivo* microscopic images during endoscopy, enabling to make real-time diagnosis, and to perform targeted biopsies improving the diagnostic accuracy. These newly developed technologyies have been evaluated for several diseases of different districts, such as lung and bladder [31, 32], and in particular it was suggested that pCLE can be employed in the detection of several gastrointestinal tract diseases. In fact, Wang et al. (2011) in his recent work concludes that pCLE can assess the severity of *Helicobacter pylori* gastritis [33], Meining et al. support that pCLE can be used in the management of indeterminate pancreaticobiliary structures [34], and Gaddam developed six diagnostic criteria to identify dysplasia in Barrett's esophagus [35]. However, there are conflicting advises about the CLE promising utility: in fact, Bisschops in an editorial entitled *“Confocal laser endomicroscopy: finally ready to change clinical practice?”* agreed that the CLE is an innovative imaging tool but not enough to justify its use in a general endoscopy unit [36]. Therefore, advantages and limitations of this novel imaging tool, in particular of the pCLE, need to be acknowledged.

ACF, first reported by Bird [37], are considered the result of first insult in CRC and [8], therefore, represent the putative earliest known morphological precursors to colorectal adenoma, capable of progression to CRC, and a marker of colorectal cancer risk [38–41]. However, there are still conflicting data about ACF meaning: some authors consider ACF as detectable first step of colon carcinogenesis; others do not recognize this role. They are localized colonic mucosal alterations involving crypts and their surface is epithelium. Histologically, ACF are not specific entity, but morphologically and genetically heterogeneous lesions.

ACF can be identified by high-magnification chromoendoscopy (MCE), but there is still variability in the endoscopic criteria used to define these lesions. The morphologic features most commonly used are darker staining [12–14, 18], larger crypt size [13, 14], raised appearance [13, 16, 18], thicker epithelial lining [14], and dilated crypt lumen [16], compared to the surrounding normal mucosa. In the different studies concerning ACF, a great variability in prevalence [12, 13] and correspondence to histology was found [14, 16]. In fact, data obtained in works using MCE [42] show a prevalence of ACF ranging from 15% [12] to 100% [13] in patients with a normal colon on colonoscopy and from 0% [12] to 61% [16] in patients with sporadic colorectal carcinoma, while the rate of agreement between the endoscopic identification of ACF and histological confirmation ranges from 53% [16] to 92% [14]. This may reflect the actual difficulty in identifying accurately small lesions needed to be biopsied and, more importantly, the necessity to define the endoscopic criteria of ACF [43].

Therefore, in this study we evaluated the correspondence between endoscopic identification by pCLE and histological diagnosis of a small series of ACF, to validate the promising role of pCLE as useful tool in the evaluation of colorectal preneoplastic lesions, in particular ACF. In our work, 7 out of 9 cases (78%) diagnosed as ACF on conventional endoscopy and confirmed on pCLE showed corresponding histological features.

Basing upon our results, we consider a real advantage of the potential of obtaining microscopic images in real time using pCLE because it allows an accurate endoscopic diagnosis and a contemporaneous possibility of treatment, with corresponding time savings and reduced costs of the procedure. Moreover, the “*in vivo diagnosis*” could significantly reduce the number of biopsies to be performed, restricting to those lesions with a real malignant potential, for example, in the management of several chronic diseases, and so limiting the adverse reactions that could occur during multiple randomized biopsy [30]. pCLe major disadvantages are operator dependency regarding the difficulty in maintaining the stability of the probe and in the interpretation of morphologic features and the limited depth of penetration of the tool [6]. This study was designed on the basis of results obtained in two previous works, in which we reported our experience in the identification of superficial colonic neoplasia and in DALM associated with CUC [4, 5]. In fact, we first demonstrated that pCLE has a predictive value of *in vivo* identification of colorectal preneoplastic and neoplastic lesions [4]. In the second one, instead, we showed the switch from the inflamed to neoplastic mucosa in a patient with chronic ulcerative colitis (CUC) [5]. We confirmed previously reported data, according to Kuiper's work [44], in which they also proposed a new pCLE colon classification, highlighting the high level of accuracy of pCLE in identifying colonic intraepithelial neoplasia. Furthermore, we demonstrated the clinical applicability and predictive power of pCLE also in a group of aberrant colonic crypt foci (ACF) collected during laser confocal endomicroscopy, through the concordance between endoscopic and histological features (rate of architectural alterations in the absence or presence of epithelial dysplasia).

## 5. Conclusion

To the best of our knowledge, this work constitutes the first attempt to correlate the identification of ACF by pCLE with the actual putative advantages, both in terms of final diagnosis and the concern of the compliance of patients for the endoscopy procedure. Although this study considered only a limited number of patients, the results obtained allow us to suppose that this endoscopic image technique is extremely useful in the identification of these putative very early colonic preneoplastic lesions. This leads to several considerations: the introduction of this imaging technique in an endoscopy unit allows to save time, decreasing both the risk for patients during colonoscopy and the procedure's costs. These data will be validated in future studies on a significantly larger study population. However, these preliminary findings support the idea that pCLE may significantly improve our chances to morphologically specifically detect the colon areas corresponding to ACF, thus increasing the diagnostic accuracy.

## References

[1] ISD online http://www.isdscotland.org/isd/1696.html.

[2] Jiang XB, Yang QY, Sai K, Zhang XH, Chen ZP, Mou YG (2011). Brain metastases from colorectal carcinoma: a description of 60 cases in a single Chinese cancer center. *Tumor Biology*.

[3] Brenner H, Bouvier AM, Foschi R (2011). The EUROCARE Working Group. Progress in colorectal cancer survival in Europe from the late 1980s to the early 21st century: the EUROCARE study. *submitted to International Journal of Cancer*.

[4] De Palma GD, Staibano S, Siciliano S (2010). *In vivo* characterisation of superficial colorectal neoplastic lesions with high-resolution probe-based confocal laser endomicroscopy in combination with video-mosaicing: a feasibility study to enhance routine endoscopy. *Digestive and Liver Disease*.

[5] De Palma GD, Staibano S, Siciliano S (2011). *In-vivo *characterization of DALM in ulcerative colitis with high-resolution probe-based confocal laser endomicroscopy. *World Journal of Gastroenterology*.

[6] Buchner AM, Shahid MW, Heckman MG (2010). Comparison of probe-based confocal laser endomicroscopy with virtual chromoendoscopy for classification of colon polyps. *Gastroenterology*.

[7] Hoffman A, Goetz M, Vieth M, Galle PR, Neurath MF, Klesslich R (2006). Confocal laser endomicroscopy: technical status and current indications. *Endoscopy*.

[8] Kinzler KW, Vogelstein B (1996). Lessons from hereditary colorectal cancer. *Cell*.

[9] Gupta AK, Pinsky P, Rall C (2009). Reliability and accuracy of the endoscopic appearance in the identification of aberrant crypt foci. *Gastrointestinal Endoscopy*.

[10] Corpet DE, Tache S (2002). Most effective colon cancer chemopreventive agents in rats: a systematic review of aberrant crypt foci and tumor data, ranked by potency. *Nutrition and Cancer*.

[11] Das P, Jain D, Vaiphei K, Wig JD (2008). Abberant crypt foci—importance in colorectal carcinogenesis and expression of p53 and mdm2: a changing concept. *Digestive Diseases and Sciences*.

[12] Huristone DP, Karajeh M, Sanders DS, Drew SK, Cross SS (2005). Rectal aberrant crypt foci identified using high-magnification-chromoscopic colonoscopy: biomarkers for flat and depressed neoplasia. *American Journal of Gastroenterology*.

[13] Rudolph RE, Dominitz JA, Lampe JW (2005). Risk factors for colorectal cancer in relation to number and size of aberrant crypt foci in humans. *Cancer Epidemiology Biomarkers and Prevention*.

[14] Takayama T, Katsuki S, Takahashi Y (1998). Aberrant crypt foci of the colon as precursors of adenoma and cancer. *New England Journal of Medicine*.

[15] Yokota T, Sugano K, Kondo H (1997). Detection of aberrant crypt foci by magnifying colonoscopy. *Gastrointestinal Endoscopy*.

[16] Adler DG, Gostout CJ, Sorbi D, Burgart LJ, Wang L, Harmsen WS (2002). Endoscopic identification and quantification of aberrant crypt foci in the human colon. *Gastrointestinal Endoscopy*.

[17] Lopez-Ceron M, Pellise M (2012). Review article: biology and diagnosis of aberrant crypt foci. *submitted to Colorectal Disease*.

[18] Schlemper RJ, Riddell RH, Kato Y (2000). The Vienna classification of gastrointestinal epithelial neoplasia. *Gut*.

[19] Norlida AO, Phang KS (2010). Histomorphology of aberrant crypt foci in colorectal carcinoma. *Malaysian Journal of Pathology*.

[20] Di Gregorio C, Losi L, Fante R (1997). Histology of aberrant crypt foci in the human colon. *Histopathology*.

[21] Fenoglio-Preiser CM, Noffsinger A (1999). Aberrant crypt foci: a review. *Toxicologic Pathology*.

[22] Lambert R, Lightdale CJ (2003). The Paris endoscopic classification of superficial neoplastic lesions: esophagus, stomach, and colon. *Gastrointestinal Endoscopy*.

[23] Roncucci L, Stamp D, Medline A, Cullen JB, Bruce WR (1991). Identification and quantification of aberrant crypt foci and microadenomas in the human colon. *Human Pathology*.

[24] Roncucci L, Medline A, Bruce WR (1991). Classification of aberrant crypt foci and microadenomas in human colon. *Cancer Epidemiology Biomarkers and Prevention*.

[25] Pretlow TP, Barrow BJ, Ashton WS (1991). Aberrant crypts: putative preneoplastic foci in human colonic mucosa. *Cancer Research*.

[26] Pretlow TP, O’Riordan MA, Pretlow TG, Stellato TA (1992). Aberrant crypts in human colonic mucosa: putative preneoplastic lesions. *Journal of Cellular Biochemistry*.

[27] Wallace M, Lauwers GY, Chen Y (2011). In process citation. *Endoscopy*.

[28] Yeung TM, Mortensen NJ (2011). Advances in endoscopic visualization of colorectal polyps. *Colorectal Disease*.

[29] Paull PE, Hyatt BJ, Wassef W, Fischer AH (2011). Confocal laser endomicroscopy: a primer for pathologists. *Archives of Pathology and Laboratory Medicine*.

[30] van den Broek FJC, van Es JA, van Eeden S (2011). Pilot study of probe-based confocal laser endomicroscopy during colonoscopic surveillance of patients with longstanding ulcerative colitis. *Endoscopy*.

[31] Wiesner C, Jäger W, Salzer A (2011). Confocal laser endomicroscopy for the diagnosis of urothelial bladder neoplasia: a technology of the future?. *British Journal of Urology International*.

[32] Thiberville L, Salaün M (2010). Bronchoscopic advances: on the way to the cells. *Respiration*.

[33] Wang P, Ji R, Yu T (2010). Classification of histological severity of *Helicobacter pylori*-associated gastritis by confocal laser endomicroscopy. *World Journal of Gastroenterology*.

[34] Meining A, Chen YK, Pleskow D (2011). Direct visualization of indeterminate pancreaticobiliary strictures with probe-based confocal laser endomicroscopy: a multicenter experience. *Gastrointestinal Endoscopy*.

[35] Gaddam S, Mathur SC, Singh M (2011). Novel probe-based confocal laser endomicroscopy criteria and interobserver agreement for the detection of dysplasia in barrett’s esophagus. *American Journal of Gastroenterology*.

[36] Bisschops R (2011). Confocal laser endomicroscopy: finally ready to change clinical practice?. *Gastrointestinal Endoscopy*.

[37] Bird RP (1987). Observation and quantification of aberrant crypts in the murine colon treated with a colon carcinogen: preliminary findings. *Cancer Letters*.

[38] Khare S, Chaudhary K, Bissonnette M, Carroll R (2009). Aberrant crypt foci in colon cancer epidemiology. *Methods in Molecular Biology*.

[39] Mutch MG, Schoen RE, Fleshman JW (2009). A multicenter study of prevalence and risk factors for aberrant crypt foci. *Clinical Gastroenterology and Hepatology*.

[40] Gupta AK, Schoen RE (2009). Aberrant crypt foci: are they intermediate endpoints of colon carcinogenesis in humans?. *Current Opinion in Gastroenterology*.

[41] Sakai E, Takahashi H, Kato S (2011). Investigation of the prevalence and number of aberrant crypt foci associated with human colorectal neoplasm. *Cancer Epidemiology Biomarkers and Prevention*.

[42] Gupta AK, Pretlow TP, Schoen RE (2007). Aberrant crypt foci: what we know and what we need to know. *Clinical Gastroenterology and Hepatology*.

[43] Rasheed S, Rigas B (2008). Screening for colorectal cancer: does it all start with aberrant crypt foci?. *Gastrointestinal Endoscopy*.

[44] Kuiper T, van den Broek FJC, van Eeden S (2011). New classification for probe-based confocal laser endomicroscopy in the colon. *Endoscopy*.

